# Associations between symptoms with healthcare utilization and death in advanced cancer patients

**DOI:** 10.1007/s00520-023-07618-5

**Published:** 2023-02-23

**Authors:** Megan M. Farrell, Cherry Jiang, Gabriel Moss, Barbara Daly, Elizabeth Weinstein, Matthew Kemmann, Mona Gupta, Richard T. Lee

**Affiliations:** 1 Case Western Reserve University School of Medicine, 21300 Cornell Rd, Cleveland, OH 44106 USA; 2 University Hospitals, 11100 Euclid Ave, Cleveland, OH 44106 USA; 3 Cleveland Clinic, 9500 Euclid Ave, Cleveland, OH 44195 USA; 4 City of Hope Comprehensive Cancer Center, Duarte, CA USA

**Keywords:** Palliative care, Symptom burden, Quality of life, Healthcare utilization

## Abstract

**Introduction:**

There is limited data about assessments that are associated with increased utilization of medical services among advanced oncology patients (AOPs). We aimed to identify factors related to healthcare utilization and death in AOP.

**Methods:**

AOPs at a comprehensive cancer center were enrolled in a Center for Medicare and Medicaid Innovation program. Participants completed the Edmonton Symptom Assessment Scale (ESAS) and the Functional Assessment of Cancer Therapy–General (FACT-G) scale. We examined factors associated with palliative care (PC), acute care (AC), emergency room (ER), hospital admissions (HA), and death.

**Results:**

In all, 817 AOPs were included in these analyses with a median age of 69. They were generally female (58.7%), white (61.4%), stage IV (51.6%), and represented common cancers (31.5% GI, 25.2% thoracic, 14.3% gynecologic). ESAS pain, anxiety, and total score were related to more PC visits (B=0.31, 95% CI [0.21, 0.40], *p*<0.001; B=0.24 [0.12, 0.36], *p*<0.001; and B=0.038 [0.02, 0.06], *p*=0.001, respectively). Total FACT-G score and physical subscale were related to total PC visits (B=−0.021 [−0.037, −0.006], *p*=0.008 and B=−0.181 [−0.246, −0.117], *p*<0.001, respectively). Lower FACT-G social subscale scores were related to more ER visits (B=−0.03 [−0.53, −0.004], *p*=0.024), while increased tiredness was associated with fewer AC visits (B=−0.039 [−0.073, −0.006], *p*=0.023). Higher total ESAS scores were related to death within 30 days (OR=0.87 [0.76, 0.98], *p*=0.027).

**Conclusions:**

The ESAS and FACT-G assessments were linked to PC and AC visits and death. These assessments may be useful for identifying AOPs that would benefit from routine PC.

## Introduction

Oncology patients in advanced stages of their disease typically experience multiple symptoms of varying severity. These range from adverse effects of oncologic treatments and/or the underlying disease process itself. Fatigue, pain, insomnia, and weight loss are common symptoms in cancer patients and are often not consistently addressed by clinical teams in treatment [1]. These symptoms have a negative impact on the quality of life, cost of care, and survival rates [2]. Therefore, there is growing interest in early incorporation of palliative care interventions for cancer patients.

Palliative care (PC) refers to an approach that aims to improve the quality of life of patients with severe illnesses through physical, psychosocial, and spiritual approaches to symptom relief [3]. In general, patients who are dealing with severe symptoms are not seen by palliative care providers unless by request of a physician. However, patients who are referred to palliative care are often referred late and have worse symptom burden [4]. Part of the reason patients who need palliative care interventions are not receiving them in a timely manner is that there has been no established standard for assessing and prompting a palliative care referral in oncology [5].

Several studies have shown that patient-reported outcomes (PROs) have clinical importance. For example, symptom monitoring with PROs has been correlated with improved health-related quality of life (HRQL), fewer ER visits, increased overall survival, and higher quality-adjusted survival rate [6, 7]. Therefore, consistently using PROs, such as the Edmonton Symptom Assessment Scale (ESAS), could help in monitoring symptom burden and therefore improve outcomes among cancer patients. This measure may also correlate with various predictors of symptom burden, including race, cancer location, comorbidity, and number of hospital admission. A few studies have assessed symptom burden of advanced cancer patients over a prolonged period and healthcare utilization [8–11]. The aim of the present study is to explore the association of symptom burdens with medical utilizations and death in a population of advanced oncology patients enrolled in a prospective supportive care intervention. Specifically, we hope to assess for physiological, psychological, and social factors and symptoms related to medical utilizations and death in advanced cancer patients.

## Methods

### Patients

In total, 1331 patients participated in the study and were enrolled in the Learning Individual Needs and Coordinating Care (LINCC) program, and 817 patients had adequate data for analysis. LINCC was an outpatient program at University Hospitals Seidman Cancer Center (SCC) funded by the Center for Medicare and Medicaid Innovation (CMMI) program (1C1CMS331349) [12]. The eligibility criteria for this program were: (1) ≥18 years old; (2) Medicare or Medicaid beneficiary; and (3) advanced or progressed solid tumor malignancy. Participants were identified from the LINCC team by several methods including: attending tumor boards, reviewing providers’ schedules, and referrals from the clinical teams. From 2014 to 2018, the CMMI program connected adult patients with primarily advanced solid tumor cancers to early palliative care interventions. The goal of this program was to improve quality of care, quality of life, patient experience, and reduce cost of care. This would be achieved by improving care coordination, proactively assessing biopsychosocial needs, clarifying patient goals and values, routinely involving specialty palliative care, and testing an innovative payment model. Prior to this program at SCC, no routine biopsychosocial screening or PROs were done, nor was there consistent discussion of goals of care or advanced planning. The reasons varied for why a subset of patients had longitudinal assessments including lost to follow-up and death.

Nurse care coordinators from the CMMI program were assigned to patients and conducted regular biopsychosocial assessments with their assigned patients. Nurse care coordinators administered the ESAS and distress thermometer at least monthly, and all assessments at baseline, 30 days, 90 days, and 180 days until the program ended, the patient dropped out of the program, or the patient expired. Assessments that were administered during the program included the ESAS, distress thermometer, Functional Assessment of Cancer Therapy-General (FACT-G), Functional Assessment of Chronic Illness Therapy-Spiritual Well-Being (FACIT-Sp), and Patient-Reported Outcomes Measurement Information System (PROMIS). Nurse coordinators reviewed the responses from patients and then provided follow-up as clinically indicated including contacting the primary oncology team and palliative care providers. Our current study focused on the responses to the ESAS and FACT-G assessments due to redundancy of the measures with the PROMIS and limited response to the FACIT-Sp assessments due to a change of assessment tool during the study.

Demographic data collected included age at enrollment date, sex, race, type of health insurance, location of treatment, tumor site, original cancer stage, and treatment (surgery, systemic cancer therapy, radiation) during the period of enrollment. Location, comorbidity score, number of admissions, number of ER visits, type of insurance, and death were obtained from billing and electronic medical records as well as recorded by the nurse care coordinators during the duration of the program.

### Validated outcome measures

#### ESAS

The Edmonton Symptom Assessment Scale (ESAS) is a validated and reliable measure used to identify the prevalence and severity of nine symptoms commonly associated with cancer patients [13–15]. Each symptom is rated on a scale of 0 (*none*) to 10 (*worst possible*). ESAS scores can be commonly assigned to the following categories: mild symptoms (1–3), moderate symptoms (4–6), and severe symptoms (7–10) [16]. A totaled ESAS score (out of 90) gives a measure of the overall symptom burden of a patient with higher scores indicating worse symptoms.

#### FACT-G

The Functional Assessment of Cancer Therapy-General (FACT-G) is a validated measure containing 27 questions about physical well-being, social/family well-being, emotional well-being, and functional well-being [17, 18]. Responses for each item range from 0 (*not at all*) to 4 (*very much*). The FACT-G assessment provides total scores for four subscales: physical well-being, social/family well-being, emotional well-being, and functional well-being. Higher scores indicate a better quality of life.

### Comorbidity

The Centers for Medicare and Medicaid Services (CMS) uses a hierarchical condition category (HCC) risk adjustment model to calculate risk scores [19]. This model categorizes diagnoses based on similar cost patterns. Chronic conditions that contribute to higher predicted healthcare costs result in higher risk scores. We used HCC scores as a measure of comorbidity in our analysis.

### Medical services utilization and death

We evaluated patients’ medical services utilization based on their total number of PC, acute care (AC), and emergency room (ER) visits while enrolled in the program as well as if the patient died during their enrollment. AC refers to a specific clinic available at the cancer center for urgent medical issues that did not require ER level care. For example, a patient with nausea and dehydration but with stable vital signs could be seen in the AC clinic rather than the ER to receive IV medications and intravenous fluids. This data was collected primarily from billing and electronic medical records.

### Statistical analysis

Our aim was to explore and identify physiological, psychological, and social factors and symptoms related to medical utilizations and death in advanced cancer patients. We utilized the self-report and medical data from patients enrolled in the LINCC program. The data spans a total of 21 months. We conducted all analyses using IBM SPSS Statistics Version 26.0. To understand our sample breakdown and characteristics, we conducted frequency and descriptive analyses (mean, SD) of our sample and demographic information (e.g., age, gender, tumor site).

We explored symptoms related to palliative care utilizations by conducting a series of multiple linear regressions, with total number of palliative care visits as the dependent variable. We examined different models based on total ESAS and FACT-G assessment scores, FACT-G subscales, ESAS physical dimensions (e.g., pain), and ESAS psychological dimensions (e.g., anxiety). For the models examining predictors of PC visits, we used a mean of the aggregate assessment responses across time points for each patient. Other medical utilization included the total number of AC and ER visits. Our analyses for these additional medical utilization outcomes were the same as those we used to examine symptoms and PC utilizations, where we conducted an analysis for total assessment scores, FACT-G subscale scores, ESAS physical items, and ESAS psychological items. We controlled each model for multicollinearity, patient demographic factors, medical services other than the outcome variable of the model, and number of days per month for each medical service utilization. We conducted the analyses using a stepwise enter method.

Finally, to analyze symptoms related to death, we conducted a series of logistic regressions, with death (yes/no) as the outcome variable. We used the ESAS and FACT-G assessments as our predictors. Specifically, we included total ESAS and total FACT-G scores in the model in order to examine if these assessments were related to death. Another model included each FACT-G subscale as the predictor variables of interest in order to examine if any of these scales and their associated symptom categories were related to death. We created models examining the individual, physical items of the ESAS assessment (e.g., pain), as well as a model examining the individual psychological items of the ESAS assessment (e.g., anxiety) as our predictor variables of interest in order to examine if there are any specific symptoms related to death. We ran each model on patient responses 0–30 days before death, 31–90 days before death, 91–180 days before death, and more than 1 year before death. We controlled each model for multicollinearity, demographic factors of the patients, medical utilizations, and utilization per month. We conducted the regression analyses using a stepwise enter method.

## Results

### Patient characteristics

Patients’ ages ranged from 19 to 97 years of age (*M*= 67.3, *SD*= 12.7). The majority of patients were female (58.7%) and white (61.4%). Most patients were treated at the main campus (an academic tertiary care center) (79.9%). Patients’ cancer types included a range of cancers, including gastrointestinal (31.5%), thoracic (24.8%), gynecologic (14.3%), head/neck (13.1%), and breast (5.7%). Most patients enrolled in the program were diagnosed as stage III (29.1%) or IV (51.6%). Treatments that patients received included systemic cancer therapy (50.8%), radiation therapy (32.3%), and surgery (27.5%) (see Table 1).

**Table 1:** Patient characteristics

Characteristic	N (%)
Age	
<50	86 (10.5)
51–60	117 (14.3)
61–70	245 (29.7)
71–80	266 (32.3)
>80	103 (12.5)
Sex	
Male	332 (40.2)
Female	(485 (58.7)
Race	
White	507 (61.4)
Non-white	296 (35.8)
Unknown	14 (1.7)
Location treated	
Main Campus	660 (80.8)
Community campus	157 (19.2)
Tumor site	
Breast	47 (5.7)
GI	260 (31.5)
Gynecologic	118 (14.3)
Head/neck	108 (13.1)
Thoracic	205 (24.8)
Other	79 (9.6)
Initial cancer stage	
Precancerous	1 (0.1)
Stage I	28 (3.4)
Stage II	70 (8.5)
Stage III	240 (29.1)
Stage IV	426 (51.6)
Unknown	52 (6.3)
Treatment received*	
Systemic cancer treatment	420 (50.8)
Radiation	267 (32.3)
Surgery	227 (27.5)
Died	
Yes	504 (61.0)
No	313 (37.9)

* indicates that total percentage exceeds 100%, as treatment type was not mutually exclusive

### Predictors of medical utilization

#### Palliative care visits

##### ESAS

Total ESAS scores were related to palliative care visits (B= 0.03, 95% CI [0.02, 0.06], *p*= 0.001) indicating that for every 1-unit increase in patients’ total ESAS scores, patient’s total palliative care visits increased by 0.03 visits. Among the physical symptoms, only pain was significantly related to palliative care visits (B= 0.31 [0.21, 0.40], *p*< 0.001). Among our sample, for every 1-unit increase in pain scores, total palliative care visits increased by 0.31 visits. No other physical symptoms were statistically related to palliative care visits. Among the psychological symptoms, only anxiety was related to palliative care visits (B= 0.24 [0.12, 0.36], *p*< 0.001). Thus, among our sample, for every 1-unit increase in anxiety ratings, total palliative care visits increased by 0.24 visits. Depression was not related to palliative care visits (see Table 2).

**Table 2:** Predictors of total palliative care visits

	B	CI 95%	*P*
ESAS total score	**0.038**	**[0.017, 0.059]**	**0.001**
Pain	**0.305**	**[0.212, 0.398]**	**<0.001**
Tiredness	0.07	[−0.045, 0.186]	0.231
Nausea	−0.024	[−0.139, 0.092]	0.689
Depression	0.025	[−0.102, 0.151]	0.703
Anxiety	**0.239**	**[0.115, 0.363]**	**<0.001**
Drowsiness	0.006	[−0.129, 0.140]	0.933
Appetite	0.034	[−0.076, 0.143]	0.547
Well-being	0.007	[−**0**.115, 0.129]	0.910
Shortness of breath	0.029	[−0.064, 0.122]	0.540
FACT-G total score	**−0.021**	**[−0.037, −0.006]**	**0.008**
Physical Subscale	**−0.181**	**[−0.246, −0.117]**	**<0.001**
Social subscale	0.017	[**−**0.042, 0.076]	0.572
Emotional subscale	**−**0.043	[**−**0.113, 0.026]	0.222
Functional subscale	0.005	[**−**0.060, 0.070]	0.889

Bolded items indicate a significant relationship

##### FACT-G

Total FACT-G scores were similarly related to total palliative care visits (B= −0.02 [−0.04, −0.006], *p*= 0.008). Since higher FACT-G scores are indicative of better total quality of life, this indicates that for every 1-unit increase in total FACT-G scores, patients’ total palliative care visits decreased by 0.02 visits. The FACT-G subscales include physical, social, psychological, and functional symptoms. Only the physical subscale was related to palliative care visits (B= −0.18 [−0.25, −0.12], *p*< 0.001). The physical subscale of the FACT-G assessment is reverse coded so that higher scores reflect better pain outcomes. Thus, among our sample, for every unit increase in pain score, total palliative care visits decreased by 0.18 visits. No other subscales were related to total palliative care visits.

#### Acute care and emergency room visits

##### ESAS

Among the physical symptom items of the ESAS questionnaire, only tiredness was related to total acute care visits (B= −0.04 [−0.07, −0.006], *p*= 0.023). Thus, in this sample, for every 1-unit increase in patients’ tiredness ratings, total acute care visits decreased by 0.04 visits. No other physical item was related to acute care visits nor were FACT-G scores. For ER visits, only, shortness of breath (dyspnea) was related (B= −0.04 [−0.08, −0.003], *p*= 0.033). For every 1-unit increase in shortness of breath ratings, we found that ER visits decreased by 0.04. None of the other physical items was significantly related to ER visits, though tiredness approached significance (B= 0.045, *p*= 0.064).

##### FACT-G

Total FACT-G scores were not significantly related to total ER visits, but among the subscales, the social subscale was related to total ER visits (B= −0.03 [−0.05, −0.004], *p*= 0.024). Thus, among our sample, for every 1-unit increase in patients’ social support ratings, the total number of ER visits decreases by .03 visits (see Table 3).

**Table 3:** Associations with medical utilization

	AC Visits			ER Visits		
	B	CI 95%	P	B	CI 95%	*P*
ESAS total score	0.003	[−0.004, 0.009]	0.422	0.003	[−0.006, 0.012]	0.485
Pain	−0.004	[−0.032, 0.024]	0.774	0.011	[−0.029, 0.050]	0.595
Tiredness	−**0.039**	**[**−**0.073,** −**0.006]**	**0.023**	0.045	[−0.003, 0.092]	0.064
Nausea	0.009	[−0.025, 0.043]	0.589	0.044	[−0.003, 0.092]	0.067
Depression	0.021	[−0.014, 0.057]	0.240	0.044	[−0.006, 0.095]	0.087
Anxiety	−0.01	[−0.045, 0.025]	0.581	-0.021	[−0.071, 0.029]	0.411
Drowsiness	0.001	[−0.038, 0.041]	0.944	0.0001	[−0.055, 0.055]	0.998
Appetite	0.012	[−0.02, 0.044]	0.458	0.011	[−0.034, 0.056]	0.625
Well-being	0.02	[−0.016, 0.056]	0.270	−0.043	[−0.093, 0.007]	0.092
Shortness of breath	0.023	[−0.004, 0.050]	0.097	−**0.042**	**[**−**0.08, -0.003]**	**0.033**
FACT-G total score	0.0005	[−0.004, 0.005]	0.847	0.002	[−0.004, 0.009]	0.458
Physical subscale	−0.002	[−0.022, 0.018]	0.832	0.01	[−0.017, 0.038]	0.460
Social subscale	0.003	[−0.016, 0.021]	0.786	−**0.028**	**[**−**0.053,** −**0.004]**	**0.024**
Emotional subscale	0.0003	[−0.022, 0.021]	0.986	0.006	[−0.024, 0.035]	0.705
Functional subscale	−0.001	[−0.021, 0.019]	0.924	0.004	[−0.023, 0.031]	0.779

Bolded items indicate a significant relationship

#### Death

##### ESAS and FACT-G

Of the 817 subjects, 504 died during follow-up. We first examined if total ESAS or FACT-G scores were related to death within 30 days prior to date of death (DoD). Only total ESAS scores were related to death within 30 days (OR= 1.16, *p*= 0.027). This indicates that among our sample, for every 1-unit increase in total ESAS scores within 30 days of DoD, patients were 1.16 times more likely to die. The FACT-G subscales, ESAS physical symptom items, ESAS psychological symptom items were not related to death within 30 day, 31–90 days, 91–180 days, or >1 year prior to death.

## Discussion

Our goal was to explore the symptom burden of advanced oncology patients who were enrolled in a prospective supportive care intervention program. Specifically, we sought to identify the physical, psychological, and social factors and symptoms related to AOP’s medical utilization and death. In fact, we found various specific symptoms that are related to medical utilization, as well as total scores from the ESAS and FACT-G assessments. Specifically, symptoms such as pain and anxiety were related to more palliative care visits. We also found that the ESAS assessment was associated with patient death within 30 days of DoD. A few unique findings include that increased social support as measured by the FACT-G were associated to fewer total ER visits. Additionally, increased fatigue and shortness of breath we correlated with decreased AC and ER visits, respectively. We also evaluated total hospital admissions and days spent in the hospital but did not find any significant findings. The results of this study help support a standard approach for physicians to evaluate which patients could benefit from a palliative care referral.

Total ESAS and FACT-G scores were significant predictors of palliative care utilizations among the patients included in our sample. Specifically, we found that patients with higher total ESAS and/or lower FACT-G scores were more likely to have palliative care visits. As expected, the physical subscale of the FACT-G assessment was significantly related to palliative care utilization, where patients with lower total physical scores on the FACT-G assessment utilized palliative care more often. Additionally, the specific physical symptom of pain and the psychological symptom of anxiety were related to palliative care utilizations, where patients who reported higher burdens of pain and/or anxiety also utilized palliative care more. A study among advanced lung cancer patients with depression found increased enrollment in hospice, although lower likelihood of other healthcare utilization [10]. Taken together, these results suggest that early ESAS and FACT-G assessments may be helpful in identifying patients who would benefit from palliative care. Specifically, patients with higher total ESAS and/or FACT-G scores may be ideal patients for palliative care referrals as well as those with higher pain or anxiety. Of note, this study did not assess for all types of palliative visits, specifically inpatient palliative are consultations. Additionally, these finding should be taken within in the context of the entire LINCC program which included nurse coordinators that often involved referrals to the palliative care team.

These findings are especially important given previous findings that palliative care, especially early interventions of palliative care, help with patients’ symptom burdens, improve overall quality of life, increase the rate of survival, lower overall costs of treatments and care, and decrease overall health system utilizations [20–23]. If physicians are able to identify patients who match the profile of someone who would benefit from palliative care with a quick and simple tool, such as the ESAS or FACT-G assessments, this could allow physicians to refer patients to palliative care relatively early in their cancer treatment. This, in turn, could potentially improve patient quality of life and potentially overall survival. In fact, the ESAS tool has been used in clinical settings to monitor patients already in a palliative care setting or clinic [15, 24, 25]. Other studies have also found symptom assessments with the ESAS and NCCN Distress Thermometer to be associated with increased healthcare utilization among cancer populations [11, 26]. The results of the current study suggest that the ESAS tool may be useful for not simply monitoring patients already receiving palliative care, but in actually identifying patients who would benefit from palliative care.

Two symptoms were related to acute care utilizations by AOP patients—fatigue and dyspnea. Specifically, patients who reported more severe tiredness were less likely to utilize AC clinic and those with dyspnea visited the ER less. These findings are in contradiction to other studies in regard to these specifics symptoms [11, 26]. This unexpected finding may suggest that when patients were feeling particularly tired or dyspneic, they lacked the energy or motivation to receive help. In fact, previous research has found that patients often site not feeling well as a reason for missing or canceling scheduled appointments [27–30]. However, any conclusions drawn from these results are speculative, as delving into the reasons behind the relationships between variables is beyond the scope of the current study.

We found a significant relationship between the FACT-G social subscale and patients’ total ER visits. Specifically, as patients’ ratings of social support increased, their total number of ER visits decreased. This corroborates previous studies that have found a link between social support and medical utilizations. For example, Kouzis and Eaton found that patients who did not have a strong social network increased use of health services [31]. Additionally, higher social support has been linked to better health literacy, fewer health utilizations, and better overall community health [32–34]. Thus, the results of the current study suggest that the social subscale of the FACT-G assessment may be particularly useful for identifying patients who may be at-risk for higher rates of medical utilizations, such as ER visits and thus prompt earlier referral to social services.

We found a relationship between patients’ total ESAS scores and death within 30 days of DoD. This supports previous research that examined the ESAS tool as a prognosticator of patients with cancer, showing that symptom scores on the ESAS assessment deteriorate proportionately as patients get closer to death [15, 35]. Specifically, previous studies have found that symptom scores on the ESAS assessment deteriorate proportionately as patients get closer to death. Although the current study found that total ESAS scores predicted death, other studies have found that patients’ ratings of the individual items of dyspnea, lack of appetite, drowsiness, fatigue, and depression were especially reflective of patients nearing death [36]. Thus, ESAS may be useful for identifying patients who may be nearing the end stages of their illness, and thus would benefit from palliative care services. We did not find any predictors of death beyond the 0–30-day interval. This is similarly in accordance with previous studies that examined the use of the ESAS assessment as a prognosticator of cancer patients, which only found a relationship between the assessment and death within a few weeks of death (same citations as above). Nevertheless, most studies examine the use of the ESAS tool in patients’ final days. More research in the use of the ESAS to predict death beyond within 30 days of DoD is needed to determine if this tool may be useful for predicting death at longer intervals.

### Limitations

We obtained our sample and data through a tertiary academic cancer center, which included two community sites. Furthermore, the patient population from which our data were gathered included only those who have advanced cancer and have either Medicare or Medicaid insurance. Thus, our sample and subsequent results are limited in generalizability to a wider cancer patient population. Our data also relied on patient PROs, which makes our data vulnerable to response biases by the patients. Patients may have believed that responding a certain way to the measurements would influence their cancer care. Additionally, if a patient was particularly tired, short on time, or received caregiver help in answering the questions, then their responses may not have accurately represented their symptoms. A large proportion of the studies enrolled in the LINCC program had missing data and thus were not able to be included in the analysis, and no information was collected to determine why patients were lost to follow-up. Furthermore, nurses involved in the CMMI program collected the PRO data given this was a pragmatic demonstration project, rather than research assistants. However, there was no standardized method for data collection, which likely introduced some noise and variance into our data. For instance, only a small percentage of patients had a breast cancer diagnosis. Finally, this analysis was retrospective in nature and prospective studies are needed.

## Conclusion

In this cohort of advanced oncology patients, the ESAS and FACT-G assessments were associated with palliative care, acute care, and ER utilization. Specific symptoms of pain, anxiety, and physiological symptoms were most significant in predicting higher palliative care visits. Surprisingly, fatigue and dyspnea were associated with decreased AC and ER visits. Higher social support was also related to reduced ER visits. Finally, higher total ESAS scores were associated with death within 30 days. These results provide the basis for a comprehensive symptom profile of advanced cancer patients who may benefit from palliative care, or who may be at risk for higher medical utilizations or death. Physicians may be able to effectively use these tools to find and monitor patients who would benefit from palliative care referrals or who may be at risk for death in the near future. Whether use of PROs alone versus PROs together with nurse coordinators and early palliative care involvement would have similar outcomes needs to be considered and would require additional prospective randomized clinical studies, which really addresses the fact that identifying patients’ needs alone is inadequate unless it can be addressed. Furthermore, this study does not address a more important issue of how to integrate early palliative care for AOP that is sustainable for community cancer centers in a clinically effective manner, a topic being addressed by many cancer organizations including the American Society of Clinical Oncology [37].

## Data Availability

All survey data available for review.
